# Designing synthetic RNAs to determine the relevance of structural motifs in picornavirus IRES elements

**DOI:** 10.1038/srep24243

**Published:** 2016-04-07

**Authors:** Javier Fernandez-Chamorro, Gloria Lozano, Juan Antonio Garcia-Martin, Jorge Ramajo, Ivan Dotu, Peter Clote, Encarnacion Martinez-Salas

**Affiliations:** 1 Centro de Biologia Molecular Severo Ochoa, Consejo Superior de Investigaciones Cientificas -Universidad Autonoma de Madrid, Nicolas Cabrera 1, 28049 Madrid, Spain; 2 Biology Department, Boston College, 140 Commonwealth Ave, Chestnut Hill, MA 02467, USA; 3 Research Programme on Biomedical Informatics (GRIB), Department of Experimental and Health Sciences, Universitat Pompeu Fabra, Dr. Aiguader 88, Barcelona, Spain

## Abstract

The function of Internal Ribosome Entry Site (IRES) elements is intimately linked to their RNA structure. Viral IRES elements are organized in modular domains consisting of one or more stem-loops that harbor conserved RNA motifs critical for internal initiation of translation. A conserved motif is the pyrimidine-tract located upstream of the functional initiation codon in type I and II picornavirus IRES. By computationally designing synthetic RNAs to fold into a structure that sequesters the polypyrimidine tract in a hairpin, we establish a correlation between predicted inaccessibility of the pyrimidine tract and IRES activity, as determined in both *in vitro* and *in vivo* systems. Our data supports the hypothesis that structural sequestration of the pyrimidine-tract within a stable hairpin inactivates IRES activity, since the stronger the stability of the hairpin the higher the inhibition of protein synthesis. Destabilization of the stem-loop immediately upstream of the pyrimidine-tract also decreases IRES activity. Our work introduces a hybrid computational/experimental method to determine the importance of structural motifs for biological function. Specifically, we show the feasibility of using the software RNAiFold to design synthetic RNAs with particular sequence and structural motifs that permit subsequent experimental determination of the importance of such motifs for biological function.

RNA plays a central role in many cellular processes, acting as a major player in gene expression control^1^. In recent years, efforts in synthetic biology have produced synthetic riboregulators, capable of regulating gene expression^2^, and small conditional RNAs capable of self-assembly via the mechanism of hybridization chain reaction^3^. Using pipelines that included software Mfold [respectively, Vienna RNA Package RNAfold] to compute free energies and secondary structures of various constructs, synthetic thermoswitches [respectively, riboswitches] were constructed^4^,^5^. Synthetic type III hammerhead ribozymes were designed using the inverse folding software RNAiFold^6^ to generate sequences that fold into the consensus secondary structure of Peach latent mosaic viroid (PLMVd) type III ribozyme in Rfam database^7^. RNAiFold uses Constraint Programming, hence is the only complete inverse folding algorithm, i.e., it can calculate all the sequences that fold into the target structure or prove that none exists^8^. RNA design strategy using RNAiFold involves the generation of hundreds of thousands or millions of sequences that fold into a given target structure, followed by the application of various computational filters to prioritize the best candidates for experimental validation^9^. An overview of synthetic RNA design using RNAiFold 2.0 is given in^8^, which software supports numerous additional design constraints, such as allowing nucleotide sequence constraints, requiring the sequence to code for certain amino acids, requiring solutions to be compatible with another given secondary structure (in addition to folding into the target structure), allowing the specification of a list of prohibited base pairs, etc.

In eukaryotic mRNAs, a variety of mechanisms have been proposed to allow the ribosomal machinery to recognize the translation start codon. These are the scanning of the 5′UTR following cap-dependent recruitment of the translation machinery, the cap-independent mechanisms guided by the m^6^A modification of the mRNA, the RNA looping, and the direct entry driven by Internal Ribosome Entry Site (IRES) elements^10^,^11^,^12^. In the cap-dependent mechanism, recruitment of a 40S ribosomal subunit to an mRNA is facilitated by eukaryotic initiation factors (eIFs) interacting with the m(7)G cap and/or poly(A) tail, such that protein synthesis starts at the initiation codon placed near the 5′ end of the mRNA. In contrast, IRES elements, which were originally discovered in the genomic RNA of picornaviruses^10^, govern protein synthesis using a cap-independent mechanism, ignoring AUG codons near the 5′end of the mRNA. Thus, picornavirus IRES elements promote the direct recognition of an internal AUG codon located far downstream of the 5′end. Functional IRES elements have been subsequently found in other viral RNAs^13^ and in a subset of cellular mRNAs^14^,^15^,^16^. However, despite the fact that they perform the same function, there is a lack of overall conserved features among the different classes of IRES elements described so far^17^.

The RNA sequences and secondary structures of picornaviruses show higher heterogeneity than in hepatitis C virus (HCV). Accordingly, the picornavirus IRES elements are grouped into five different types (I, II, III, IV and V); each type harbors conserved sequence motifs and a common RNA structure core maintained by evolutionarily conserved substitutions^18^,^19^. In addition, each type has distinctive factor requirements. Type I is present in the genome of enterovirus, while type II is present in the genome of encephalomyocarditis virus (EMCV) and foot-and-mouth disease virus (FMDV). The activity of both type I and type II IRES depends on both the RNA structural organization and the interaction with host factors, in such a way that the assembly of translation initiation factors (eIFs) and RNA-binding proteins (RBPs) on the IRES molecule is dictated by the RNA structural architecture^19^.

Picornavirus IRES elements are organized in modular domains. In type I and type II IRES, each domain consists of one or more stem-loops that provide the binding site for RBPs and various eIFs, with the exception of eIF4E^20^,^21^,^22^,^23^. The structure of type II IRES elements is arranged in domains designated 2 to 5 (see Fig. 1A); domain 5 consists of a short hairpin followed by a single-stranded stretch of nucleotides at its 3′end including a conserved pyrimidine tract (Py); this domain provides the binding site for several proteins including eIF4B, and PTB, among other RNA-binding proteins^24^,^25^. Although all picornavirus IRES elements are capable of internal recruitment of the ribosomal subunits, little is known about the events that allow the recognition of the initiator AUG following 40S recognition in the different types of picornavirus IRES elements.

Experimental evidence supports the involvement of RNA structural motifs for IRES activity^26^,^27^,^28^. In particular, the conserved Py tract located on the distal 3′ region of the picornavirus IRES^29^ has been reported as an accessible, essential motif of type II IRES elements^24^,^30^,^31^,^32^. In the case of the FMDV IRES, the deletion of the UUUCCUU motif or its substitution to UGUGGUG abolished IRES activity^33^,^34^. Moreover, mutations affecting the Py tract interfere simultaneously with the formation of initiation complexes and the interaction with polypyrimidine tract binding protein (PTB)^35^. The PTB binding sites on the IRES elements of EMCV and FMDV RNAs have the consensus sequence CUUU and are located near the 5′ and 3′ borders of the IRES^36^, consistent with the role of PTB in IRES-mediated initiation to stabilize a specific active conformation^32^.

RNA viruses in general, and FMDV in particular, are characterized by a high genetic variability^37^. This feature, however, does not affect every position of the genome to the same extent. As it occurs in many RNA regulatory elements, evolutionary conserved motifs involved in IRES activity preserve RNA secondary structure in addition to short stretches of nucleotide sequence. The Py tract of picornavirus IRES elements belonging to type I and II tolerates some variations in the order of U/C residues^38^,^39^. In contrast, there is high sequence variability within the region that separates the Py tract from the first functional AUG codon, a feature that led to propose that this region was a spacer. However, both the length and the structure of the spacer region could contribute to ensure recognition of the authentic initiator codon by the translation machinery^22^,^40^.

In spite of the mutational analysis carried out in the picornavirus IRES Py tract, it remains elusive whether having the Py tract in a unique structural conformation is an absolute requirement for IRES activity. To answer this question we have designed candidate RNA sequences adopting different conformations of domain 5, but harboring a pyrimidine tract of the same length as that of wild type IRES. Hence, the pyrimidine tract could be either unpaired or base-paired in stem-loops with different stability. For this, we have made use of the FMDV IRES, a type II IRES element whose secondary structure is well characterized^41^ to construct synthetic RNA domains capable of adopting different structures within domain 5, at the distal 3′end of the IRES element. Domain 5 consists of three structural motifs: a hairpin, a pyrimidine-rich tract and a variable sequence (Fig. 1A). The hairpin has been described as the binding site of eIF4B^25^,^42^, while the Py tract provides the binding site for PTB^35^. It should be noted that both, the hairpin and the pyrimidine tract are strongly conserved among field isolates, whereas the spacer region shows high sequence variability (Fig. 1B). Taking advantage of this feature, novel subsets of IRES elements were generated by replacing the wild type sequence with the computationally designed RNA element fused to the luciferase open reading frame sequence. Functional and structural analysis of these elements provided information on the relationship between the accessibility of the Py tract and the structure of the hairpin of domain 5 with IRES activity. Thus, our study shows that a strategy based on *in silico* design can be successful in constructing complex functional RNA elements.

## Results

### Design of RNA structural elements sequestering the pyrimidine tract

**Figure 1:**
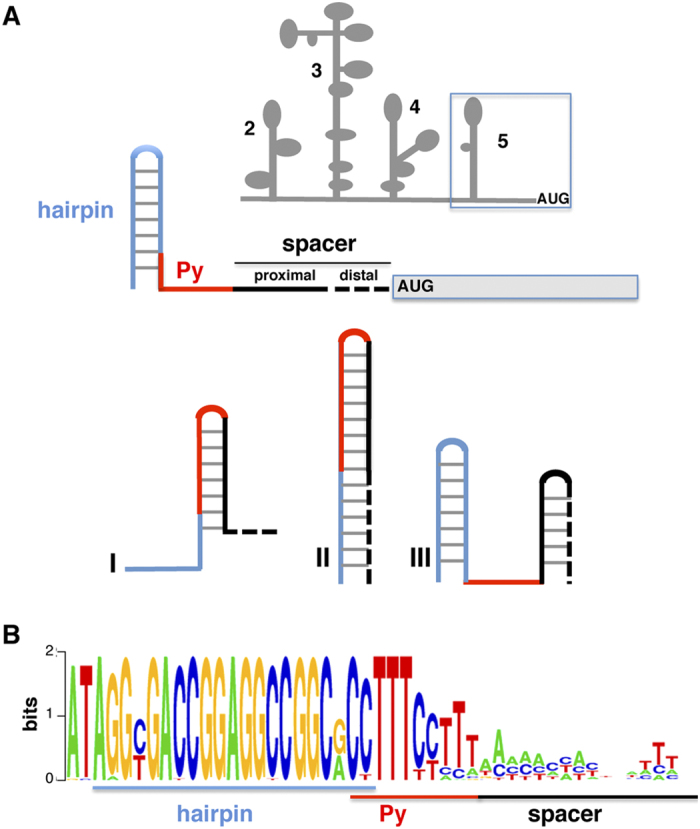
(**A**) Design strategy for synthetic RNAs harboring distinct conformations of the pyrimidine tract. (Top) Schematic representation of the FMDV IRES, organized in domains 2, 3, 4, and 5. Diagram of the structural motifs of the wild type domain 5: a hairpin (blue line), pyrimidine tract (Py) (red line) and a spacer (black line), including a proximal and a distal sequence, upstream of the functional start codon (AUG) of the reporter gene. (Bottom) Design of RNA families I, II or III. (**B**) The pattern of nucleotide conservation (measured in bits) of domain 5 (nts 417–462) is represented by a sequence logo obtained from the alignment of the FMDV IRES sequences of field isolates deposited in data banks.

To design RNA structural motifs with unique folding patterns, potentially affecting initiation of protein synthesis, we focused our attention on domain 5 of the FMDV IRES, located immediately upstream of the functional translation initiation codon (Fig. 1A). Modification of the RNA structure of this domain could provide hints about the relevance of the pyrimidine tract accessibility, as well as the potential drawback of stable stem-loops for internal initiation of translation.

The experimentally determined RNA structure of domain 5 consists of three elements (Fig. 1A), of which the hairpin and the pyrimidine tract are strongly conserved among field isolates. The last element, considered to be a spacer (filled black line in Fig. 1A, proximal spacer), shows high sequence variability (Fig. 1B). Insertion of a non-viral sequence immediately downstream of this region (distal spacer, dashed black line in Fig. 1A) is compatible with IRES activity^22^,^40^. Hence, we took advantage of the variability of the proximal spacer and the permissiveness of the distal spacer to design RNA candidates having different locations of the hairpin, while maintaining the pyrimidine tract sequence at the same position with respect to the wild type IRES sequence (Fig. 1A, bottom panel).

**Figure 2:**
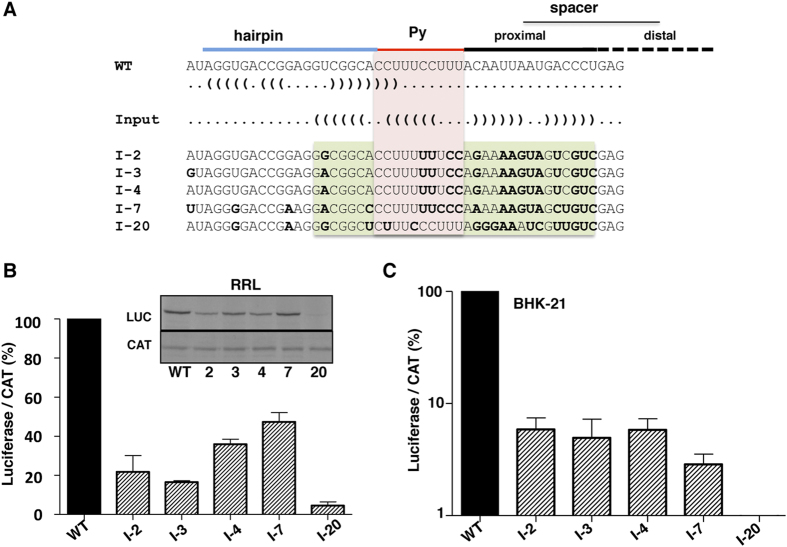
(**A**) Alignment of sequences belonging to family I candidates with the sequence of wild type (WT) domain 5. Blue, red and black lines depict the position of the hairpin, the pyrimidine tract and the spacer sequences, respectively. The RNA structure of domain 5 and the input for RNAiFold are shown in dot bracket notation. Bold letters denote the sequence changes relative to the wild type IRES sequence. A pink box denotes the location of the pyrimidine tract, while a green box depicts the residues predicted to form a hairpin in the selected candidates. (**B**) *In vitro* synthesized bicistronic RNAs (200 ng) bearing the WT or the candidate I-2, I-3, I-4, I-7, or I-20 sequences were used to program translation in rabbit reticulocyte lysates (RRL) during 60 min at 30 °C. ^35^S-labeled proteins were resolved in 12% SDS-PAGE. An autoradiogram of a representative assay is shown in the insert. The intensity of ^35^S-labeled luciferase and CAT polypeptides was measured in a densitometer; the ratio of luciferase/CAT was calculated and then normalized to the intensity observed in the WT RNA, which was set to 100%. Values correspond to the mean (±SD) of three assays. (**C**) Relative IRES activity was determined in transfected BHK-21 cells as the ratio of luciferase to chloramphenicol acetyl-transferase expressed from bicistronic constructs carrying the candidate sequences, normalized to the activity observed for the wild-type IRES (set to 100%). Each experiment was performed in triplicate and repeated at least three times.

The target structure used as input to RNAiFold^6^ is shown in Fig. 2A. In principle, no sequence constraints were imposed in the inverse folding pipeline for the proximal spacer region, given the large sequence variability of this region. However, specific Py tract nucleotides that are conserved and might be relevant for IRES function independent of its structure were fixed in the design. Supplementary Fig. S1A shows the input file to RNAiFold. RNAiFold generated a large number of sequences that fold into the target structure, subsequently filtered by Boltzmann probability of forming the target structure. Next, several measures (see Methods) were calculated in order to prioritize candidates for experimental validation. Supplementary Table S1 shows the list of all the sequences whose probability of target structure formation exceeded 0.20, along with values for other measures considered. The *in silico* design and posterior filtering process produced sequences with high probability of having the Py base-paired. Among these sequences (which were arbitrarily named), sequence I-20 was selected because it had the highest probability for the Py tract to be base-paired. Moreover, four additional sequences (I-2, I-3, I-4 and I-7) with moderate and low probability among the filtered sequences were selected in order to later establish correlations between measures and IRES activity. Measures shown to have high correlation with IRES activity can then be used as optimization criteria in subsequent design rounds.

As devised, the selected candidates (RNA family I) had a pyrimidine tract of the same length as the wild type RNA (Fig. 2A), and U/C residues were randomly selected by the inverse folding pipeline. Moreover, the region corresponding to the hairpin of domain 5 in family I of RNA candidates, containing minimal primary sequence substitutions, was predicted to be unpaired in all cases. In contrast, substitutions on the proximal spacer attempted to generate a hairpin by forcing base pairing between the pyrimidine tract and the proximal spacer sequences (Fig. 2A). All the candidates of this family harbor a substitution of a non-functional AUG by UAG or UCG; substitution of this triplet by UAA did not affect IRES function nor did it modify the assembly of 48S complexes^20^. Sequences from candidates I-2, I-3, I-4 and I-7 were closely related. Specifically, I-2 and I-4 differed only at nt 432, and I-3 and I-4 at nt 417. Candidate I-7 displayed a higher degree of variability showing 6 to 7 substitutions relative to candidates I-3 and I-4, or I-2, respectively (Fig. 2A). On the other hand, candidate I-20 shared the lowest sequence identity with the rest of family I. It should be noted that none of the candidates selected by the RNA design approach were similar to any sequences found in natural isolates; indeed, comparative analysis of natural and selected candidate sequences indicated that family I candidates form a separate group in a phylogenetic tree, independent of all field isolates (Supplementary Fig. S2).

### Functional analysis of the selected candidates

As a first attempt to analyze the influence of sequestering the pyrimidine tract within the stem of a hairpin, IRES activity of the selected candidates was determined using a cell-free system programmed with equal amounts of *in vitro* synthesized RNA. As shown in Fig. 2B, the efficiency of protein synthesis measured as the ratio of ^35^S-labeled luciferase (LUC) polypeptide to chloramphenicol acetyl transferase (CAT) polypeptide in rabbit reticulocyte lysates (RRL) was reduced in all selected candidates relative to the wild type RNA. Note that the activity of RNAs I-2, I-3, I-4, and I-7 was very similar. However, the activity of RNA I-20 was reduced to a higher extent than all other candidates (Fig. 2B). Three C:G pairs are predicted in the hairpin sequestering the Py tract in RNA I-20, while only A:U or U:G pairs are predicted in the analogous hairpin in RNAs I-2, I-3, I-4, and I-7, suggesting that the stability of this hairpin could be inversely related with the efficiency of internal initiation of translation. Similar results were obtained using monocistronic constructs, in which translation of firefly luciferase was dependent on the FMDV IRES and translation of renilla luciferase was cap-dependent (Supplementary Fig. S3).

To further reinforce the biological relevance of the selected candidates using a different system, we measured luciferase (Luc) activity expressed from bicistronic RNAs in transfected BHK-21 cells (Fig. 2C). The same extract was also used to determine chloramphenicol acetyl transferase (CAT) activity as a control of the transfection efficiency. In this assay translation of CAT reflects the efficiency of cap-dependent translation initiation, while that of luciferase reflects the activity of cap-independent translation initiation. Consistent with results from the *in vitro* experiments, the relative IRES efficiency measured as the ratio of luciferase/CAT was similar for BHK-21 cells driven by candidates I-2, I-3, I-4, and I-7, and lower than the wild type (Fig. 2C). Again, activity of RNA I-20 was clearly below all the others. Taken together, these results suggest that sequestering the Py tract exerts a negative effect on IRES activity, both *in vivo* and *in vitro*.

### Experimental RNA structure probing supports the accuracy of RNA design

**Figure 3:**
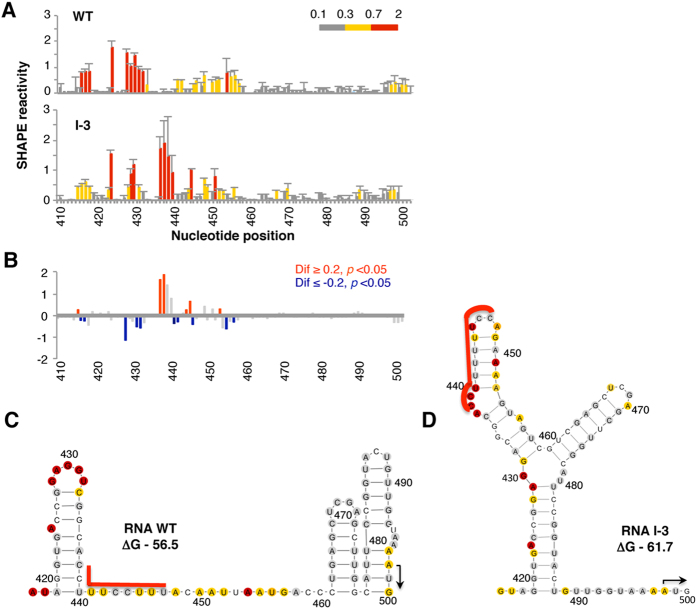
(**A**) SHAPE reactivity profiles of the wild type (WT) domain 5 and the candidate I-3 RNAs extended to the AUG start codon (nts 410–500) using fluorescent-labeled primers and capillary electrophoresis. SHAPE reactivity (mean ± standard deviation of a triplicate assay) was represented in a colored scale in which 0 indicates unreactive nucleotides and the average intensity of highly reactive nucleotides is set to 1. Nucleotide positions are indicated on the x-axis. (**B**) Significant SHAPE reactivity differences between the WT and the variant I-3 RNAs. Red or blue bars depict nucleotides with *p*-values < 0.05 and absolute SHAPE reactivity differences (Dif) higher or lower than 0.2, respectively. Grey bars depict differences which are non statistically significant. RNA structure model of the wild type domain 5 (**C)** or the candidate RNA I-3 **(D)** extended to the functional AUG codon, imposing SHAPE reactivity (colored as in A) on RNAstructure. The first predicted structure, and its minimal free energy (ΔG) value, is depicted in each case. A red line depicts the position of the Py tract. Nucleotides are numbered every 10 positions; an arrow marks the AUG start codon.

To gain information about the RNA structure of the selected candidates, we chose a representative member of the family, RNA I-3, to analyze its local RNA flexibility in solution using SHAPE methodology (Fig. 3A). SHAPE reactivity correlates inversely with the probability that a nucleotide is base-paired^43^. Structural analysis was performed using a transcript harboring the entire IRES fused to the luciferase coding region, N-methylisatoic anhydride (NMIA) as the modifying agent and fluorescent-labeled primers^41^. As a control, the wild type RNA was analyzed in parallel. The pattern of SHAPE reactivity obtained in triplicate assays for the candidate I-3 and wild type RNAs is shown in Fig. 3A. RNA I-3 displayed a pattern of NMIA accessibility that specifically differed from the wild type RNA in nts 428–457, encompassing the hairpin and the Py tract of domain 5 (see *p* values and absolute SHAPE differences in Fig. 3B), revealing a reorganization of its structure.

Using RNAstructure software to predict the secondary structure including SHAPE reactivity data for wild type RNA and candidate I-3 (Fig. 3C,D), we show that relative to the wild type RNA, RNA I-3 adopted a secondary structure in which the Py tract is base-paired with downstream nucleotides, as imposed in the inverse folding pipeline. In addition, according to this RNA structure model, the nucleotides corresponding to the wild type hairpin of domain 5 (nts 419–440) are base paired with sequences 454–459 and 480–488 in RNA I-3. We conclude that the RNA structure of candidate I-3 (Fig. 3D) matched the predicted structure imposed as input on the inverse folding pipeline (Fig. 2A), effectively sequestering the Py tract in a stable hairpin. These results validated the usefulness of RNA inverse folding to design RNAs differing in their structural organization from the sequence of interest.

### Positive correlation between the structural accessibility of the pyrimidine tract and efficiency of protein synthesis

To further prove the effect of structural accessibility of the pyrimidine tract on IRES activity, we plotted the parameters used to select final candidates of family I against the efficiency of protein synthesis measured in cell-free systems (Table 1). This data indicated a strong correlation (Spearman coefficient) between values of ProbUnpaired_PTB, PLfold_PTB, and Sample_PTB-5 (see Methods) and protein synthesis (Supplementary Fig. S4). These results are also relevant from a design point of view. The strategy outlined in^6^ and^8^ consists of generating a large number of sequences that fold into a given target structure and satisfy additional sequence and/or structural constraints, and subsequently filtering candidates with respect to different measures. This approach depends on knowing which measures are most pertinent for the design problem at hand. By selecting a few random sequences and validating them, we can determine (through correlation values) which measures are most appropriate to apply in a second round of design.

### Design of RNA elements that sequester the pyrimidine tract within long, stable hairpins

**Figure 4:**
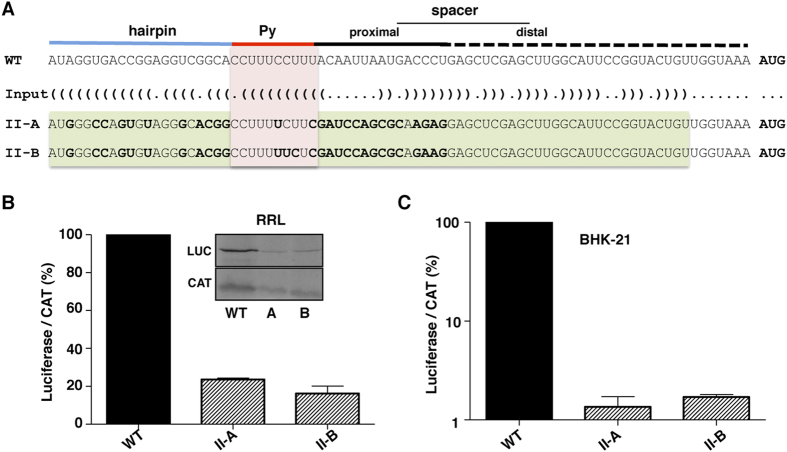
(**A**) Alignment of sequences belonging to family II candidates and the sequence of WT domain 5. The RNA structure of domain 5 and the input for RNAiFold are shown in dot bracket notation. All symbols are used as in Fig. 2A. (**B**) *In vitro* synthesized bicistronic RNAs (200 ng) bearing the wild type RNA WT or the candidates II-A or II-B were used to program translation in RRL during 60 min at 30 °C. An autoradiogram of a representative assay is shown in the insert. The intensity of ^35^S-labeled luciferase and CAT polypeptides was measured in a densitometer; the ratio of luciferase/CAT was calculated and then normalized to the intensity observed in the WT RNA, which was set to 100%. Values correspond to the mean (±SD) of three assays. (**C**) Relative IRES activity was determined in transfected BHK-21 cells as the ratio of luciferase to chloramphenicol acetyl-transferase expressed from bicistronic constructs carrying the candidate sequences, normalized to the activity observed for the wild-type IRES. Each experiment was performed in triplicate and repeated at least three times.

Since the reduced activity of RNA I-20 could be related to the higher stability of the hairpin sequestering the Py tract, we attempted to generate a second round of candidates, selected on the basis of adopting a stable hairpin that sequesters the Py tract within a long stem-loop. In order to generate sequences for this family, we again used RNAiFold with the target structure shown in Fig. 4A. In this case, our target structure takes advantage of the distal region of the spacer to create a longer and more stable stem-loop. The sequence of the distal region was fixed, and all previous considerations for family I hold in this new design (see Supplementary Fig. S1B for the input to RNAiFold). Again, we filtered the thousands of sequences returned by RNAiFold using Boltzmann probability of target structure; in this case, we only considered sequences with a probability greater than 0.02 (Supplementary Table S2). Among these, we selected two sequences (II-A and II-B) with the highest ProbUnpaired_PTB (Table 2), since it was one of the measures that had the best correlations with IRES activity.

As devised, the family II of candidates (Fig. 4A) differed from family I in the capacity to adopt a stable hairpin including the entire spacer that separates the IRES from the functional AUG codon for luciferase. Determination of IRES activity for family II members measured by *in vitro* assay indicated a strong decrease of luciferase synthesis (Fig. 4B), which was akin to that found for construct I-20. Similar results were obtained using monocistronic constructs (Supplementary Fig. S3). Also, the relative IRES activity of the constructs II-A and II-B measured as the ratio of luciferase to CAT activities determined in the same extract in BHK-21 transfected cells showed a decreased activity relative to the wild type RNA (Fig. 4C), which was similar to the results of the *in vitro* translation assays. These results suggest that sequestering the Py tract within a hairpin inactivates IRES activity; furthermore, the stronger the stability of the hairpin, the higher the inhibition of protein synthesis.

**Figure 5:**
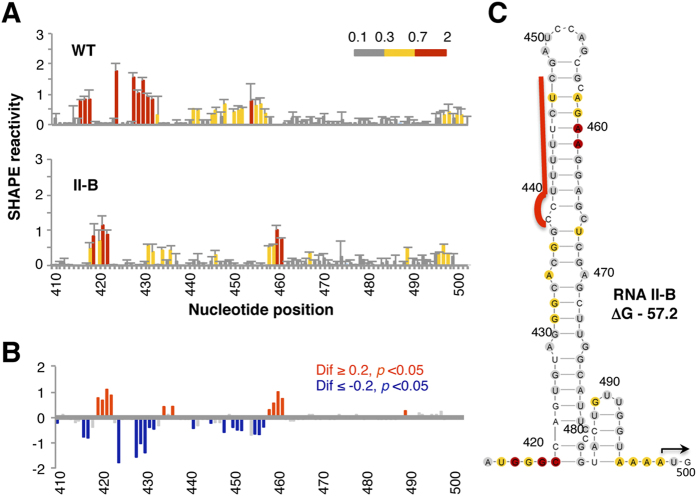
(**A**) SHAPE reactivity profiles of the wild type (WT) domain 5 and the candidate II-B RNAs extended to the AUG start codon (nts 410–500) using fluorescent-labeled primers and capillary electrophoresis. SHAPE reactivity (mean ± standard deviation of a triplicate assay) was represented in a colored scale in which 0 indicates unreactive nucleotides and the average intensity at highly reactive nucleotides is set to 1.0. Nucleotide positions are indicated on the x-axis. (**B**) Significant SHAPE differences between the WT and the variant II-B RNAs. Red or blue bars depict nucleotides with *p*-values < 0.05 and absolute SHAPE reactivity differences (Dif) higher or lower than 0.2, respectively. Grey bars depict differences which are non statistically significant. (**C**) RNA structure model of the candidate RNA II-B extended to the functional AUG codon imposing SHAPE reactivity (colored as in A) on RNAstructure. The first predicted structure, and its minimal free energy (ΔG), value is indicated. A red line depicts the position of the Py tract. Nucleotides are numbered every 10 positions; an arrow marks the AUG start codon.

Next, one candidate of family II was selected to analyze its SHAPE reactivity in solution. The pattern of SHAPE reactivity obtained in triplicate assays for candidate II-B is shown in Fig. 5A, in parallel to the wild type RNA. RNA II-B mainly differed in the pattern of reactivity from the wild type RNA in positions 416–436, 440–461 (see Fig. 5B for absolute SHAPE differences higher than 0.2 and *p* values < 0.05), confirming that the selected candidate harbored a modified RNA structure. As observed before for candidate I-3, the RNA structure model of candidate II-B obtained by imposing SHAPE reactivity on RNAstructure (Fig. 5C) resembled the structure used as input for the inverse folding pipeline (Fig. 4A), greatly differing from the wild type RNA (compare to Fig. 3C).

To further test our hypothesis, we generated the construct designated III-1 by site-directed mutagenesis (Supplementary Fig. S5A). This RNA was predicted to preserve the Py tract in an unpaired region within two hairpins; the first hairpin exactly matched the wild type, while the second hairpin occupied the spacer region. Measurement of IRES activity by *in vitro* and *in vivo* assays indicated that the RNA III-1 is almost as active as the wild type IRES element *in vitro* (Supplementary Fig. S5B), and at least 3 to 10-fold more active than any member of families I and II in BHK-21 cells. These results suggest that maintaining the Py tract in an unpaired region is an important feature for the initiation of protein synthesis, whereas modification of the structural organization of the spacer region is tolerated.

### Influence of the hairpin of domain 5 on IRES activity

**Figure 6:**
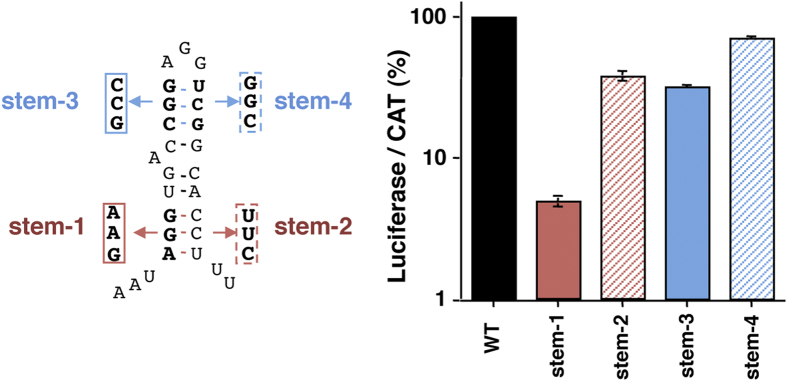
Mutational analysis of the hairpin of domain 5. (Left panel) The nucleotides substitutions inserted in mutants stem-1 or stem-3, and the compensatory substitutions in stem-2 and stem-4 are depicted in red and blue colors, respectively. (Right panel) Relative IRES activity (mean ± SD) was determined in transfected BHK-21 cells as the ratio of luciferase to chloramphenicol acetyl-transferase expressed from bicistronic constructs carrying the candidate sequence, normalized to the activity observed for the wild-type IRES (set to 100%). Each experiment was performed in triplicate wells and repeated at least three times.

The RNA structure models of variants I-3 and II-B predicted imposing SHAPE reactivity indicated that, in addition to the Py tract, the hairpin of domain 5 was reorganized. Thus, to determine whether disruption of the hairpin structure could also affect FMDV IRES activity we performed a mutational analysis on both the basal and the apical stem of the hairpin (Fig. 6, left panel). Mutations in the basal stem of the hairpin were engineered to maintain the Py tract. Disruption of the basal stem induced a reduction of IRES activity in construct stem-1, and compensatory mutations (stem-2) restored IRES function (Fig. 6, right panel). Disrupting base pairing of the apical stem modestly affected IRES activity (stem-3), although compensatory substitutions (stem-4) recovered IRES function. These data allow us to conclude that the RNA structure of the hairpin of domain 5 plays an important role on IRES activity, in agreement with its phylogenetic conservation (Fig. 1B) and with its involvement in protein interactions^25^.

## Discussion

In this study we have successfully applied a combination of *in silico, in vitro* as well as *in vivo* approaches to design modified structural motifs of the type II picornavirus IRES element in order to determine the relevance of structural accessibility of the polypyrimidine tract for initiation of protein synthesis. The RNA design strategy with synthetic RNA sequences was applied to domain 5 of the FMDV IRES element, for which the RNA structure has been determined in solution^19^. This relatively small domain consists of a hairpin followed by a pyrimidine-rich tract of about 9 nts long^33^, which provides the binding site for the protein PTB^35^, and a spacer sequence upstream of the first functional initiator codon of the viral genome. For this design, the sole requirement was that alternative structures lead to presentation or masking of the PTB-binding site. It remains elusive how other approaches might have been used to generate sequences that fold into specific structures while conserving specific nucleotides; in contrast, this is relatively straightforward by using the *in silico* design with RNAiFold, which allows exhaustive sequence generation and control over specific thermodynamic properties in the candidate selection process. The effect of the hairpin stability on IRES activity was also analyzed for the hairpin presumed to sequester the Py tract.

The inverse folding strategy represents an unbiased approach that allows exploring the sequence space for a given target RNA structure. Notably, the candidates selected by the RNA design approach do not include any sequences found in natural isolates. Indeed, phylogenetic analysis indicated that the sequences of the members of each family of candidates form a group independent of the other family, and also different from all field isolates (Supplementary Fig. S2). Since we show here that IRES elements carrying the candidate RNAs are less active than the wild type, our data could shed light on those forces in molecular evolution operating in the natural selection of IRES elements, which tend to favor the survival of active molecules to the detriment of the inactive ones.

The hierarchical folding of RNA^44^ allows modeling the secondary structure of an RNA molecule, regardless of knowing its tertiary structure. However, because of imperfect accuracy of the current prediction methods, designing a sequence that can fold into a certain conformation requires checking whether the proposed sequence actually folds into the target structure. By experimentally determining SHAPE reactivity for one representative of each family of candidates, our data strongly suggest that the selected candidates adopt a secondary structure comparable to the target structure given in the input to RNAiFold. These constructs adopt a structure different than that of the wild type domain 5, in which the hairpin of domain 5 is base-paired and the pyrimidine tract is not. Differences observed at the borders between the structure imposed in the design of the candidate RNAs and the results obtained by SHAPE for those RNAs can be explained by the fact that the structure probing experiments were performed in the context of the entire functional RNA. Given that the RNA design approach was conducted on a specific short RNA structural motif belonging to a long functional RNA, we could not discard the possibility that changes in the structure of the selected candidates could induce a distant reorganization affecting other regions of the functional element.

The structural reorganization observed in the RNA variants I-3 and II-B indicated that, in addition of embedding the Py tract on a hairpin including downstream sequences, the nucleotides corresponding to the hairpin on the wild type RNA were also reorganized. Thus, it was possible that beyond the effect of sequestering the Py tract, the decrease in IRES function could be due to the disorganization of the hairpin of domain 5. This possibility was analyzed measuring the effect of mutations that destabilize the basal or the apical base pairs of the hairpin, but conserving the Py tract. The results indicated that the secondary structure of this hairpin is important for IRES function. Thus both, sequestering of the Py tract and disorganization of the hairpin could lead to a significant change in the binding of proteins that interact with this IRES region^35^,^42^,^45^.

Although all picornavirus IRES elements recruit the ribosomal subunits internally, the events following ribosome entry differ among picornavirus RNAs. In poliovirus RNA, a representative member of type I IRES element, all ribosomes initiate translation at the AUG743, and the upstream AUG586 is ignored^46^. Thus, it is assumed that ribosomal subunits scan the poliovirus RNA from AUG586 to AUG743. In the case of the EMCV RNA, that contains a type II IRES element, the 3′ end harbors a conserved UUUC motif followed by a variable G-poor spacer. Initiation of translation starts at the 11th AUG codon from the 5′-end of the EMCV RNA, located 25 nts downstream from a conserved Py tract, ignoring the flanking AUG10 and AUG12^47^. In EMCV RNA the distance between the Py tract and the functional AUG is a critical factor. Thereby, it was concluded that the entry site is at the AUG start codon.

In contrast, in FMDV RNA, which also contains a type II IRES element, protein synthesis can start at two functional AUGs (designated AUG1 and AUG2) separated by 84 nts^48^, but only 20% of the ribosomes initiate at AUG1 and the remaining 80% at the next AUG2^21^,^22^. As in other picornavirus RNAs, upstream AUG triplets are ignored. Interestingly, a conserved pyrimidine tract is located upstream of each functional AUG codon, which in both cases is predicted to be accessible according to chemical and enzymatic RNA probing^20^. Mutations within the 84 nt inter-AUG region revealed that this sequence has no major impact on initiation of protein synthesis at AUG2, the strongest start codon.

Concerning the differences among type I and type II picornavirus IRES elements, experimental evidence points to a differential role of the spacer structure on protein synthesis. For instance, in the case of FMDV RNA, the presence of a highly stable hairpin (six repetitions of the Xma linker) within the spacer (−77 kcal/mol) but not the presence of unstructured sequences up to 99 nts corresponding to the polylinker sequence of pGem3, hinders IRES activity^40^. Hence, it was concluded that the FMDV IRES tolerated relatively long distances between the last residue of the IRES and the functional start codon, in contrast to the EMCV IRES. Previous work showed that constructs harboring the wild type IRES followed by a spacer predicted to fold as two unstable hairpins (−5.1 and −0.4 kcal/mol) (see Fig. 3B) do not interfere with IRES activity^22^. In fact, in the context of the viral RNA, the 84 nucleotide spacer that separates the functional AUG1 and AUG2 codons adopts a stem-loop structure leaving accessible the Py tract^20^.

The lack of conserved pathways to recognize start codons among different IRES elements^19^ challenges the prediction of the consequences of inserting the IRES element in different sequence contexts. Thus, our work has contributed to establish the critical role of both preserving as base-paired the stem-loop (hairpin) region of domain 5 and preserving as unpaired the pyrimidine tract for optimal IRES activity. Although a number of RBPs interact with secondary structure motifs^49^, our results are in agreement with previous data that showed specific binding of PTB to single-stranded regions of type I and type II IRES^23^,^32^,^50^. In contrast to type I and type II IRES elements, the influence of PTB on HCV IRES activity remains controversial^51^,^52^. Moreover, there are differences in the accessibility of the pyrimidine-rich sequences of the HCV IRES; indeed, while the one at the top of domain 3 is accessible to SHAPE reagents, another in domain II is mostly base-paired, and a third one is sequestered in the double pseudoknot structure^53^,^54^,^55^.

To determine potential similarities in Py accessibility of different IRES elements, we have analyzed the Rfam families of viral IRES for structural analogy. We used INFERNAL 1.1^56^ to create covariance models from pestivirus and picornavirus IRES family in Rfam and evaluated the structural homology of the sequences of other families. Among the viral IRES families, only HCV and the HCV-like (pestivirus) render e-values indicating significant similarities. However, comparison of picornavirus IRES indicated that there is similarity only between IRES elements belonging to the same type (enterovirus, type I; aphthovirus, type II; or hepatovirus, type III) (see Supplementary Table S4).

It is worth noting that expression vectors used to express more than one protein in eukaryotic cells usually contain type II picornavirus IRES elements, due to their high efficiency and their proven resistance to inhibitory conditions for cap-dependent translation. Given that spacers of variable sequences and length, often inherited from previous constructs, are found in expression vectors harboring IRES elements, our rational design provides useful data for the definition of RNA structural features of this spacer region affecting internal initiation of translation. In summary, we show here that a critical step for FMDV IRES function is the presence of an unpaired Py tract, along with the immediately upstream conserved stem-loop. All the selected candidates that form a stable hairpin sequestering the Py tract within the stem of a downstream hairpin show a significant decrease in protein synthesis despite conservation of the Py residues. To our knowledge, this is a novel finding since previous studies investigated the effect of modified Py sequences but did not investigate structural accessibility of the Py tract. Thus, future studies aimed to develop improved expression vectors should take into account the accessibility of this conserved structural motif.

Several machine learning algorithms have been developed^57^,^58^, which use either neural networks or support vector machines, in order to predict the initiator AUG start codon in eukaryotes and most recently for *H. sapiens*. A high-throughput version of the hybrid computational/experimental method described in this paper could generate sufficient data to train a regression support vector machine to predict translation efficiency of IRES elements. Such a tool could possibly provide insights concerning the ratio of protein products from genes that contain an IRES element.

## Methods

### Inverse folding Computational Pipeline

RNAiFold computes all sequences that fold into a given characteristic secondary structure^59^. Since it relies on Constraint Programming, RNAiFold is the only software that can (in principle) determine all those sequences that fold into a target structure, or determine that no solution exists. RNAiFold optionally allows the user to stipulate certain sequence constraints, such as nucleotide identities (e.g. binding site or active site), pyrimidine content, etc. that may be shared by all members of an RNA family.

Family I was designed to fold into the target structure shown in Fig. 2A. Moreover, the design constraints included fixing subsequences relevant for IRES activity, and restricting the pyrimidine tract to consist of only pyrimidine residues. The input file to RNAiFold used is shown in Supplementary Fig. S1A.

Family II was designed to fold into the target structure shown in Fig. 2B. The same design constraints were applied for this family, with the exception that the length of the spacer sequence was allowed to include the distal space in wild type IRES. The input file to RNAiFold used is shown in Supplementary Fig. S1B.

### *In silico* measures of polypyrimidine accessibility

We computationally estimated the accessibility of the polypyrimidine tract at 30 °C for each candidate using three different methods (Tables 1 and 2): First (PLfold_PTB), using RNAplfold^60^ from ViennaRNA package with the options -L 84 (length of the sequence) and -u 7 (length of Py) and extracting the value corresponding to the position of Py we obtain an estimate of the probability of having all seven positions of Py unpaired. Second (Sample_PTB-5), we sampled 100,000 low energy structures from the thermodynamic ensemble using RNAsubopt –d2 –p 100000^61^ from ViennaRNA package and computed the proportion of structures in which at least five of the seven positions of Py were unpaired. Third (ProbUnpaired_PTB), we computed the probability 1-p(i) that position i of Py is unpaired, hence the probability that at least one of the positions of Py is unpaired is 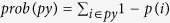.

### Constructs

Insertion of the candidate sequences into the bicistronic plasmid pBIC^26^ yielded constructs described in Supplementary Table S3. Briefly, oligonucleotides harboring the sequence of interest in positive and negative orientation were annealed in Tris 50 mM pH 7.5, NaCl 100 mM, MgCl_2_ 10 mM, 15 min at 37 °C and then inserted into the HindIII and XhoI restriction sites of pBIC, previously linearized with the same enzymes. Colonies that carried the correct insert were selected for further studies. Prior to expression analysis, the nucleotide sequence of the entire length of each region under study was determined (Macrogen).

### RNA synthesis and translation assays

*In vitro* transcription was performed for 1 h at 37 °C using T7 RNA polymerase, as described^62^. RNA was extracted with phenol-chloroform, ethanol precipitated and resuspended in TE. The integrity of the transcripts was verified by gel electrophoresis. Equal amounts of the RNAs synthesized *in vitro* were translated in 70% rabbit reticulocyte lysate (RRL) (Promega) supplemented with ^35^S-methionine (10 μCi)^45^. Each experiment was repeated independently at least three times using the wild type RNA as a control in all assays. In assays using bicistronic RNAs, the intensity of the luciferase and chloramfenicol acetyl transferase (CAT) bands produced by each transcript was determined in a densitometer, then the ratio of luc/CAT obtained for each construct was normalized against the ratio obtained for the wild type RNA in the same assay, set at 100%. Values represent the mean ± SD.

The relative IRES activity obtained in assays conducted with monocistronic RNAs (100 ng of the IRES-firefly luciferase RNA and 25 ng of the renilla luciferase RNA) was monitored in a luminometer using the dual system (Promega).

Plasmids carrying the candidate sequences upstream of the luciferase reporter gene were assayed in BHK-21 cells. Transfection of 90% confluent monolayers was carried out using cationic liposomes 1 h after infection with the Vaccinia virus VT7F-3 expressing T7 RNA polymerase. This assay excludes the presence of cryptic promoters since the transfected plasmid is transcribed in the cell cytoplasm by the T7 RNA polymerase. Extracts from 10^5^ cells were prepared 20 h after transfection in 50 μl of 50 mM Tris-HCl, pH 7.8, 120 mM NaCl, 0.5% NP40. Luciferase and chloramphenicol acetyl transferase (CAT) activities were measured as described^63^. Assays were performed in triplicate wells at least three times. Values correspond to the mean (±SD).

### SHAPE analysis

Monocistronic constructs were linearized with SphI prior to synthesis of RNA transcripts *in vitro.* RNAs (2 pmol) were treated with N-methylisatoic anhydride (NMIA)^43^ following denaturation and folding at 30 °C. Briefly, prior to NMIA treatment, *in vitro* synthesized RNA was renatured by heating at 95 °C for 2 min, snap cooling on ice for 2 min, and subsequently incubated in a final volume of 18 μl of folding mix (100 mM HEPES-KOH pH 8.0, 0.5 mM MgCl_2_, 100 mM NaCl) for 20 min at 37 °C. Then, RNA were incubated with dimethyl sulfoxide (DMSO) (untreated RNA) or 6.5 mM NMIA for 45 min at 37 °C, precipitated and resuspended in 10 μl of 0.5X TE^64^. For primer extension, 2 pmol of treated and untreated RNAs were incubated with 2 pmol of the antisense 5′-end fluorescently-labeled primer 5′-TAGCCTTATGCAGTTGCTCTCC-3′ at 65 °C for 5 min, 35 °C for 5 min and then, chilled on ice for 2 min, as described^41^. Primer extension reactions were conducted in a final volume of 16 μl containing reverse transcriptase (RT) buffer and 1 μM each dNTP. The mix was heated at 52 °C for 1 min prior to addition of 100 U of Superscript III RT and incubated at 52 °C for 30 min. A sequencing ladder was generated using the corresponding untreated RNA in the presence of 0.1 mM ddC. NED fluorophore was used for both NMIA-treated and untreated samples while FAM fluorophore was used for the sequencing ladder^64^. cDNA products were resolved by capillary electrophoresis. Electropherograms were analyzed using QuSHAPE software^65^. Triplicate assays were used to calculate quantitative SHAPE reactivity (mean ± SD) for individual data sets was normalized to a scale from 0 to 2, in which 0 indicates an unreactive nucleotide and the average intensity of highly reactive nucleotides is set to 1.0. The statistical significance of the SHAPE reactivity data obtained in the RNA variants relative to the wild type RNA was determined by the unpaired two-tail Student’s *t*-test.

### Nucleotide sequence analysis

The alignment of forty-six sequences of FMDV IRES field isolates was performed using CLUSTALX software with default parameters (http://www.clustal.org). The RNA sequences were obtained from GenBank; only domain 5 unique sequences were included in the alignment. The phylogenetic tree was inferred by the neighbour-joining method from the multiple-sequence alignment using MEGA4 program (www.megasoftware.net). Robustness of evolutionary relationships was assessed by 1000 bootstrap replicates. Bootstrap values are shown as percentages, only nodes with percentage values higher than 50% are indicated.

The sequence logo (http://weblogo.berkeley.edu) was generated from the multiple-sequence alignment of the IRES sequences. The overall height of each stack indicates the sequence conservation at that position (measured in bits), and the height of symbols within the stack reflects the relative frequency of the corresponding nucleic acid at that position.

### RNA structure modeling

Secondary RNA structure prediction accuracy can be improved incorporating SHAPE reactivity values as constraints in RNAstructure software^66^. SHAPE reactivity data is taken into account by inclusion of a pseudo-free energy parameter of [2.6 × (1+SHAPE reactivity at position i) −0.8] kcal/mol, i.e. an affine pseudo-energy contribution depending on SHAPE activity, where slope (m) is 2.6 kcal/mol and y-intercept (b) is −0.8 kcal/mol, as recommended in RNAstructure predictions^67^. The predicted structure corresponding to the lowest minimal free energy (MFE) energy was used to depict the RNA structure model. Similar RNA structure models were predicted using RNAsc^68^. Secondary RNA structure was visualized with VARNA (http://varna.lri.fr/).

## Additional Information

**How to cite this article**: Fernandez-Chamorro, J. *et al*. Designing synthetic RNAs to determine the relevance of structural motifs in picornavirus IRES elements. *Sci. Rep.* **6**, 24243; doi: 10.1038/srep24243 (2016).

## Supplementary Material

Supplementary Information

Supplementary Dataset 1

Supplementary Dataset 2

## Figures and Tables

**Table 1:** Measures of candidates in family I.

Name	Luciferase intensity	Probability of structure^a^	Ensemble defect^a^	Expected base pair distance^a^	PLfold_PTB	SamplePTB_5	ProbUnpaired_PTB
I-2	0.38	0.30	9.72	6.00	0.0994	0.148	0.486
I-3	0.28	0.32	7.87	4.81	0.0734	0.114	0.502
I-4	0.36	0.33	8.01	4.90	0.0763	0.117	0.500
I-7	0.49	0.24	10.11	5.89	0.1506	0.194	0.451
I-20	0.06	0.22	14.52	9.06	0.0000	0.000	0.569
Correlation^b^	1	0.1	0	−0.1	1	1	−1

^a^Calculated using the target structures, as described^8^.

^b^Spearman coefficient.

**Table 2:** Measures of candidates in family II.

Name	Luciferase intensity	Probability of structure	Ensemble defect	Expected base pair distance	PLfold_PTB	Sample PTB_5	ProbUnpaired_PTB
II-A	0.13	0.05	13.35	8.38	0.0159	0.021	0.976
II-B	0.09	0.05	13.34	8.38	0.0157	0.020	0.977
