# Supplementary material for: Designing synthetic RNAs to determine the relevance of structural motifs in picornavirus IRES elements
Source: Sci Rep. 2016 Apr 7;6:24243. doi: 10.1038/srep24243 (PMC4823658; doi:10.1038/srep24243)
Supplement: Supplementary Information [file srep24243-s1.pdf]

Designing synthetic RNAs to determine the relevance of structural motifs in picornavirus IRES elements

Javier Fernandez-Chamorro, Gloria Lozano, Juan Antonio Garcia-Martin, Jorge Ramajo, Ivan Dotu, Peter Clote and Encarnacion Martinez Salas

SUPPLEMENTARY FIGURES

A

```
> Py small stem
#RNAscdstr
..............((((((..((((((....))))))..))))))...
#RNAseqcon
NUNNNNNNNNNNAGGNNNNNNCNUUYYYYYYNNNNNNNNNNNNNNNGAG
#temp
30
#MAXsol
0
#dangles
2
```

B

```
> Py long stem
#RNAscdstr
((((((((((((.((((.(((.((((((((((......)).)))))))).))).)))).)))))..))).))))..........
#RNAseqcon
NUNNNNNANNNNAGGNNNNNRCYUUYYYYYYRNNNNNNNNNNNNNNGAGCUCGAGCUUGGCAUUCCGGUACUGUUGGUAAAAUG
#temp
30
#MAXsol
0
#dangles
2
```

Supplementary Figure S1. RNAiFold inputs used for the designing of candidates RNAs belonging to family I (**A**) and II (**B**). Structural and sequence constraints imposed on RNAiFold, with no limit on the number of solutions (MAXsol 0). The treatment of dangling end positions (stacked single-stranded nucleotides) corresponds to ViennaRNA Package -d 2 flag.

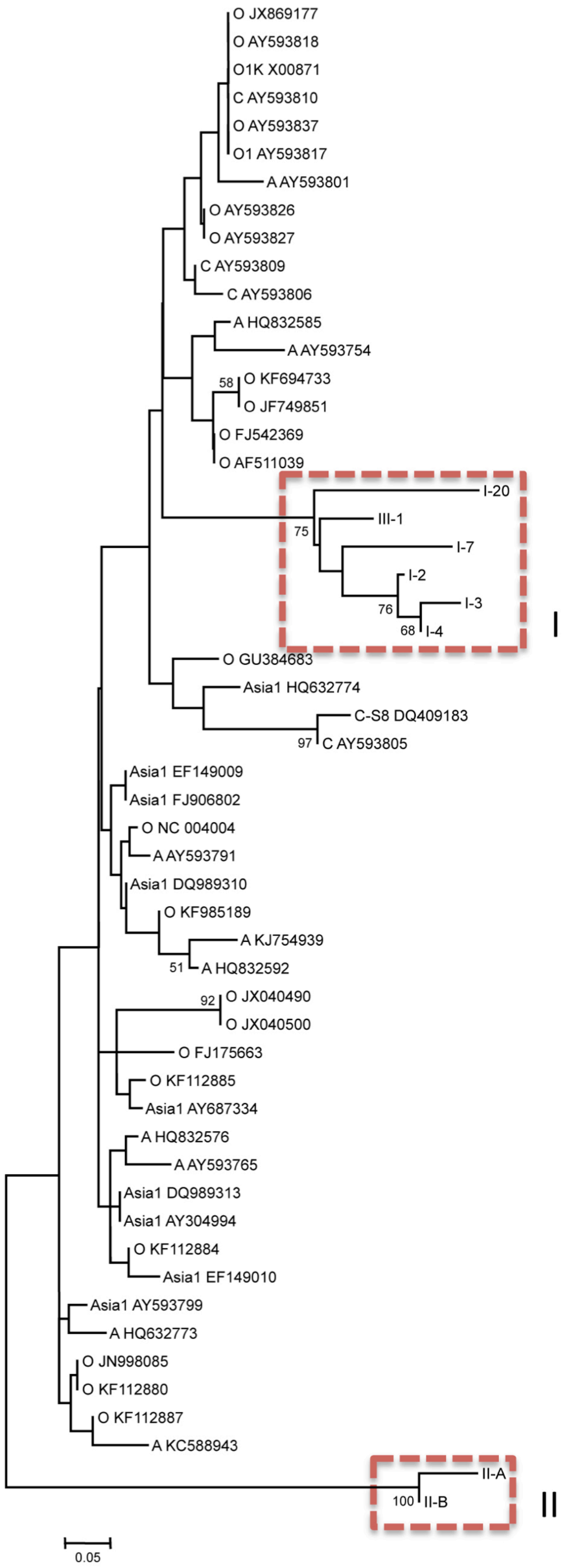

Supplementary Figure S2. Phylogenetic relationships among the nucleotide sequences of FMDV IRES domain 5 present in field isolates (GenBank accession number are indicated for each sequence) and the candidate RNAs analyzed in this work. The phylogenetic tree was

generated using the neighbour-joining method. Bootstrap values are shown as percentages, only nodes higher than 50% are labeled. The bar below the tree indicates 0.05 nucleotide substitutions per site. Dashed rectangles depict the position of the candidate RNAs.

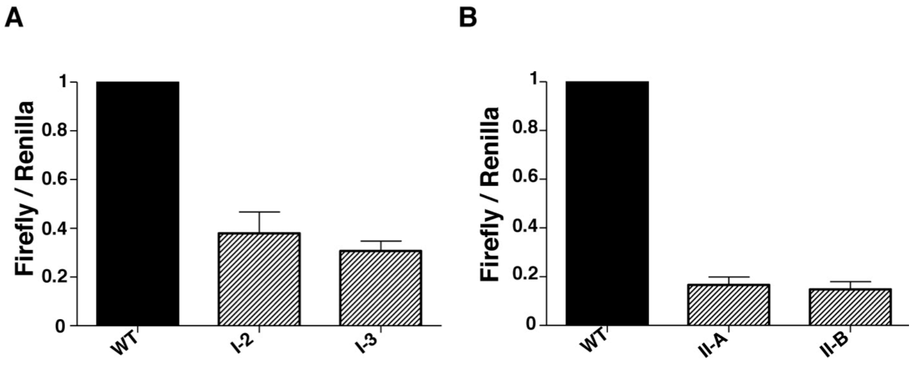

Supplementary Figure S3. Relative IRES activity determined in reticulocyte lysates using monocistronic RNAs as the ratio of firefly luciferase to renilla luciferase, relative to the activity obtained with the wt IRES. Values correspond to the mean (± SD) of three independent assays.

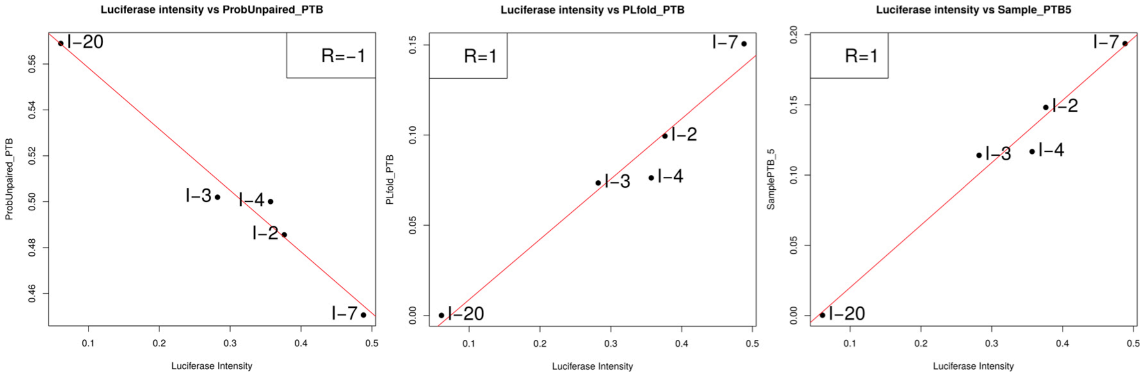

Supplementary Figure S4. Correlation between the parameters used to select the candidates of family I with translation efficiency. Plots of ProbUnpaired_PTB, PLfold_PTB and Sample_PTB-5 against the intensity of luciferase protein synthesized in *in vitro* translation assays determined for each member of the family I. The Spearman coefficient (R) is shown at the top of each panel.

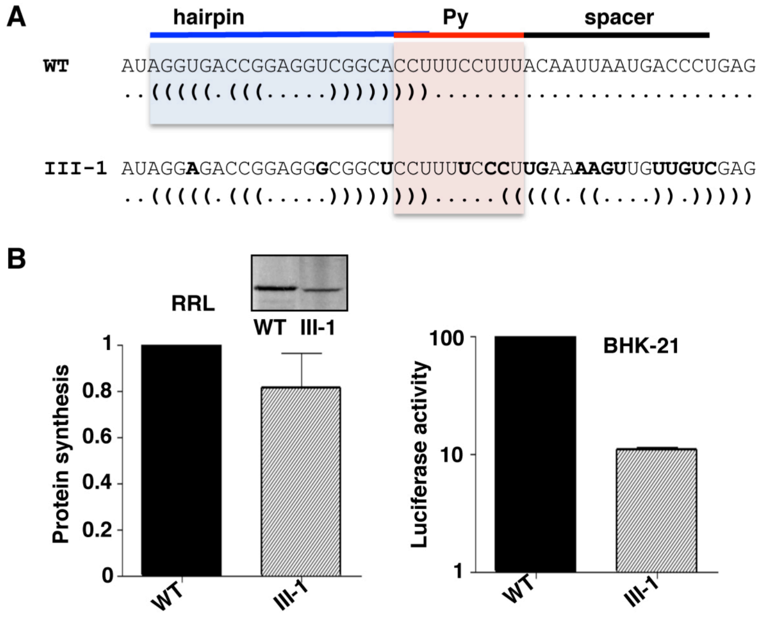

Supplementary Figure S5. A. Alignment of candidate III-1 with the sequence of domain 5. Symbols are used as in Fig. 2A. A pink box denotes the location of the pyrimidine tract, while a blue box depicts the residues predicted to form a hairpin in the wild type RNA. **B**. (Left panel) *In vitro* synthesized bicistronic RNAs (200 ng) bearing the wt or the candidate III-1 domain 5 were used to program translation in RRL during 60 min at 30ºC. An autoradiogram of a representative assay is shown in the insert. The intensity of ^{35}S-labeled luciferase was measured in a densitometer, and normalized to the intensity observed in the wt RNA, which was set to 1. Values correspond to the mean (±SD) of three assays. **(**Right panel**)** Relative IRES activity was determined in transfected BHK-21 cells as the ratio of luciferase to chloramphenicol acetyl-transferase expressed from bicistronic constructs carrying the candidate sequence, normalized to the activity observed for the wild-type IRES (set to 100%). The experiment was performed in triplicate and repeated at least three times.

Supplementary Table S3. Oligonucleotides

Name - Sequence (5´-3´)	Candidate
I-2s AGCTTCTACGCCTGAATAGGTGACCGGAGGGCGGCACCTTTTTTCCAGAAAAGTAGTCGTCGAGC	I-2
I-2as TCGAGCTCGACGACTACTTTTCTGGAAAAAAGGTGCCGCCCTCCGGTCACCTATTCAGGCGTAGA	
I-3s AGCTTCTACGCCTGAGTAGGTGACCGGAGGACGGCACCTTTTTTCCAGAAAAGTAGTCGTCGAGC	I-3
I-3as TCGAGCTCGACGACTACTTTTCTGGAAAAAAGGTGCCGTCCTCCGGTCACCTACTCAGGCGTAGA	
I-4s AGCTTCTACGCCTGAATAGGTGACCGGAGGACGGCACCTTTTTTCCAGAAAAGTAGTCGTCGAGC	I-4
I-4as TCGAGCTCGACGACTACTTTTCTGGAAAAAAGGTGCCGTCCTCCGGTCACCTATTCAGGCGTAGA	
I-7s AGCTTCTACGCCTGATTAGGGGACCGAAGGACGGCCCCTTTTTCCCAAAAAAGTAGCTGTCGAGC	I-7
I-7as TCGAGCTCGACAGCTACTTTTTTGGGAAAAAGGGGCCGTCCTTCGGTCCCCTAATCAGGCGTAGA	
I-20s AGCTTCTACGCCTGAATAGGGGACCGAAGGGCGGCTCTTTCCCTTTAGGGAAATCGTTGTCGAGC	I-20
I-20as TCGAGCTCGACAACGATTTCCCTAAAGGGAAAGAGCCGCCCTTCGGTCCCCTATTCAGGCGTAGA	
II-As AGCTTCTACGCCTGAATGGGCCAGTGTAGGGCACGGCCTTTTCTTCGATCCAGCGCAAGAGGAGC	II-A
II-Aas TCGAGCTCCTCTTGCGCTGGATCGAAGAAAAGGCCGTGCCCTACACTGGCCCATTCAGGCGTAGA	
II-Bs AGCTTCTACGCCTGAATGGGCCAGTGTAGGGCACGGCCTTTTTCTCGATCCAGCGCAGAAGGAGC	II-B
II-Bas TCGAGCTCCTTCTGCGCTGGATCGAGAAAAAGGCCGTGCCCTACACTGGCCCATTCAGGCGTAGA	
I-20s AGCTTCTACGCCTGAATAGGGGACCGAAGGGCGGCTCTTTCCCTTTAGGGAAATCGTTGTCGAGC	I-20
I-20as TCGAGCTCGACAACGATTTCCCTAAAGGGAAAGAGCCGCCCTTCGGTCCCCTATTCAGGCGTAGA	
III-1s AGCTTCTACGCCTGAATAGGAGACCGGAGGGCGGCTCCTTTTCCCTTGAAAAGTTGTTGTCGAGC	III-1
III-1as TCGAGCTCGACAACAACTTTTCAAGGGAAAAGGAGCCGCCCTCCGGTCTCCTATTCAGGCGTAGA	III-1

Supplementary Table S4. Covariance analysis of structural similarities among Rfam viral IRES families

RfamID	RF00061	RF00209	RF00210	RF00228	RF00229
RF00061					
HCV	1E-20	8.98652E-06	1.260336957	0.360434783	1.11326087
RF00209					
Pestivirus	0.272851444	1.75359E-45	0.917629348	0.340869565	0.982608696
RF00210					
Aphthovirus	0.532025316	0.5924	1.42609E-36	1.09173913	0.540434783
RF00228					
HAV	0.434556962	0.482	0.797717391	5.24443E-74	0.439782609
RF00229					
Picornavirus	1.090253165	1.204	1.467173913	0.20226087	9.87826E-40
